# Champions to enhance implementation of clinical and community-based interventions in cancer: a scoping review

**DOI:** 10.1186/s43058-024-00662-0

**Published:** 2024-10-22

**Authors:** Joseph Astorino Nicola, M. Muska Nataliansyah, Maria A. Lopez-Olivo, Adebola Adegboyega, Kelly A. Hirko, Lou-Anne R. Chichester, Nora L. Nock, Pamela Ginex, Shannon M. Christy, Paul Levett

**Affiliations:** 1 Department of Sociology, Washington State University, 310 NW Joe Street, Pullman, WA 99163 USA; 2 The genius Loci Collaboratory, Pullman, USA; 3 Department of Surgery, Division of Surgical Oncology, Collaborative for Healthcare Delivery Science, Medical College of Wisconsin, Milwaukee, WI USA; 4 Department of Health Services Research, The University of Texas MD Anderson Cancer Center, Houston, TX USA; 5 University of Kentucky College of Nursing, Lexington, KY USA; 6 Department of Epidemiology and Biostatistics, College of Human Medicine, Michigan State University, East Lansing, MI USA; 7 Memorial Sloan Kettering Cancer Center, New York, NY USA; 8 Department of Population and Quantitative Health Sciences, School of Medicine, Case Western Reserve University, Cleveland, OH USA; 9 School of Nursing, Stony Brook University, Stony Brook, NY USA; 10 Department of Health Outcomes and Behavior, Moffitt Cancer Center, Tampa, FL USA; 11 Morsansi College of Medicine, University of South Florida, Tampa, FL USA; 12 School of Medicine, George Washington University, Washington, DC USA

**Keywords:** Champion, Clinical champion, Community champion, Change agent, Implementation leader, Facilitation, Outreach, Promotion, Implementation science, Implementation strategy, Cancer

## Abstract

**Background:**

Champions are integral across research in cancer, yet studies exploring their roles are limited and have produced mixed results. The current review examines and synthesizes descriptions of how champions emerged and the types of activities they most often performed. By examining evidence from across the translational research continuum, this scoping review aims to characterize the role of champions and strategies that facilitate their involvement in the implementation of cancer care interventions in both clinical and community-based settings.

**Methods:**

This scoping review was designed and implemented in compliance with PRISMA-ScR. The review focused on peer-reviewed articles in English-language journals. We searched five databases: PubMed (including MEDLINE), Scopus (including EMBASE), CINAHL, PsycINFO, and the Cochrane Library. Articles published from 1971 to 2022 were included. Two members of the team reviewed in duplicate each article and then a single member of the team extracted the data in Covidence, with a second member comparing the extraction to the original article. Qualitative and quantitative data were extracted and then synthesized. These data were used to summarize core champion activities and implementation strategies and to characterize barriers and facilitators to using champions in research.

**Results:**

A total of 74 articles were included in the review. The qualitative synthesis highlighted facilitators and barriers to the effective use of champions. Facilitators included consideration of an individual’s characteristics when identifying champions, time spent planning for the specific responsibilities of champions, working within a supportive environment, and identifying champions embedded in the target setting. Major barriers included constrained time, low self-efficacy among champions, inadequate training, high turnover rates of champions, and a lack of buy-in from organizational leadership toward the intervention. Champions also were mostly assigned their roles, had varied core activities, and used complementary strategies to empower their target populations. Champions’ most frequent core activities include facilitation, outreach/promotion, and recruitment of participants into studies.

**Conclusions:**

Champions were used in research of many cancer types and often serve similar roles regardless of where they are located within the translational research process. Despite their critical role, evidence is lacking on the impact of champions specifically on outcomes of many of the research studies that include them. Future research is needed to understand the nuances of champion-driven approaches across diverse cancer care settings.

**Supplementary Information:**

The online version contains supplementary material available at 10.1186/s43058-024-00662-0.

Contributions to the literature • The concept of champion has evolved over time and is also not used in a standardized manner, making research on these individuals difficult

• Champions most common core activities include facilitation, outreach/promotion, and recruitment of participants into studies.

• Champions are also a cornerstone to other complementary implementation strategies within cancer care interventions.

• Champions play a critical role in ensuring the success of implementation by creating a climate for change, mitigating barriers to implementation, increasing the intervention’s reach, and expanding clinical awareness about the intervention.

## Background

 A seminal article in 1963 galvanized an academic movement to focus on individuals as a source of innovation in organizations [1]. The original concept of champions was essentially “product champions,” representing a tactic to dilute resistance to change within a single organization [1]. These champions were often the source of the innovation, acting as “inventors” within quickly growing organizations unwilling to provide support for change. For many decades little was written about champions, but between 2007 and 2012 a focus on the personal characteristics of champions within healthcare emerged [2–4].

Champions are now a common foundational implementation approach in translational research across the entire health services enterprise [5]. Champions play a role in both adoption of technologies and introduction of new behavioral patterns [6]. Champions also serve critical roles across the implementation timeline in many cancer care settings, including playing central roles in intervention delivery, as components of implementation strategies, and as part of the inner context influencing implementation [7–9]. In some cases, cancer care champions are internal to the organization [4]; in others, they are external partners or members of the wider community [10]. Despite champions being integral to innovation and change management for decades, researchers have rarely focused comprehensively on these individuals within the field of implementation science, especially their role in translational cancer research. By drawing parallels between Schon’s 1963 historical insights and contemporary challenges in implementation science, we underscore the timeless relevance of champions in facilitating organizational adaptation.

In 2020, the Cancer Consortium for Implementation Science, a public association hosted through the National Cancer Institute focused on developing new approaches and areas of investigation, identified community participation in implementation science as an important focus area for action [11]. From here, several key gaps were identified regarding the lack of knowledge about best practices for early engagement of community participants and how to identify and activate effective champions for organizational change [11]. This focus not only aligns with broader public health goals but also emphasizes the essential role of community involvement in advancing cancer care and prevention strategies.

Findings from several reviews identify the characteristics of successful champions and their overall effectiveness in facilitating implementation of change [1, 5, 6, 12, 13]. A literature review of ten articles singularly focused on champions, not confined to the cancer care literature, revealed two pathways for champions to become involved in intervention implementation. Sometimes, an individual is selected and appointed into their champion role [3, 5, 10]. More commonly, a champion emerges organically [1, 3, 5–7, 12, 14]. However, this information has not been synthesized to inform the best approach for identifying and activating champions as a formal implementation strategy.

The current review examines and synthesizes descriptions of how champions emerged, and the types of activities often performed by champions within cancer care and prevention strategies. This scoping review of the literature aims to characterize the role of champions and strategies that facilitate their involvement in the implementation of cancer care interventions in both clinical and community-based settings. Specifically, we sought descriptions of how to create the conditions for champions to emerge and what types of activities are most often performed by champions. Addressing this evidence gap is crucial; synthesizing existing evidence could unlock new strategies to enhance the effectiveness of cancer interventions, offering a roadmap for leveraging champions to achieve broader public health objectives.

## Methods

### Protocol and registration

This scoping review was designed and implemented to be in compliance with the PRISMA Extension for Scoping Review (PRISMA-ScR) [15]. The team prepared a protocol before starting the review and published this in the Open Science Framework [16].

### Eligibility criteria

Champions often bridge various settings, including research, practice, and program contexts. This wide range of evidence is indicative of the multifaceted nature of champions’ roles. A scoping review, by design, is equipped to synthesize information from varied study designs, making it an appropriate method for capturing the extensive and complex roles of champions in cancer care interventions. Therefore, we included studies with randomized and non-randomized interventions and observational designs (i.e., cohort studies, cross-sectional studies, case-control studies, prevalence studies, case series, case reports, and economic evaluations) as well as studies using mixed methods and qualitative designs. Translational research interventions were included that contained community or clinical champions within the cancer care continuum (i.e., prevention, screening, treatment, survivorship/rehabilitation). Non-English language studies, dissertations/theses, diagnostic accuracy tests, text/opinion pieces, clinical prediction rules, conference proceedings, trial information, study protocols, and preprints were excluded. Only articles published after 1971 were included, because a seminal article identifies 1971 as the date used in prior reviews of the champion concept in health services [14]. Systematic and other reviews were used to frame the background and guide the hand-searching of articles but were not analyzed as part of the review.

### Information sources

A librarian, P.L., searched the following five databases on September 9, 2022: PubMed, which includes the content of MEDLINE; Scopus, which includes the content of EMBASE; the CINAHL index of nursing and allied health literature; PsycINFO; and the Cochrane Library. Within the Cochrane Library, we specifically searched the Cochrane Database of Systematic Reviews (CDSR), Cochrane Central Register of Controlled Trials (CENTRAL), and the Clinical Answers database.

### Search

The terms for the search strategy were matched to those identified by Wood et al. [13] in their systematic review on the role of clinical champions in drug and alcohol treatment and mental health settings. We identified the National Library of Medicine (NLM) Medical Subject Headings (MeSH) for cancer prevention, risk reduction behavior, early detection, cancer therapy, survivorship, or psychological adaptation. Psychological adaptation is a MeSH term the NLM uses instead of the psychological term *adjustment* as an analog for coping behavior, skills, and strategies. The Polyglot search translator was used to replace the MeSH field code with the INDEXTERMS field code for searching Scopus, and the APA Thesaurus was used to translate MeSH terms into PsycINFO search terms. The PubMed strategy was used to identify the keywords used for the Cochrane Library strategy. Only articles published after 1970 were reviewed. The search strategies are shown in Supplementary File 1.

### Selection of sources of evidence

Screening occurred in two stages according to the eligibility criteria using Covidence [17]. Seven reviewers (P.G., J.A., M.M.N., K.A.H., S.M.C., L.-A.R.C, and A.A.) screened the titles and abstracts for relevance; each was independently screened by two reviewers. Citations that received two “yes” votes were judged relevant and carried forward to full-text screening. Disagreements were resolved through consensus or by a third-party adjudicator chosen from the initial list of seven reviewers. Full texts of citations deemed relevant were obtained and uploaded. Six reviewers (P.G., J.A., M.M.N., A.M.L.-O., L.R.C., and A.A.) screened full text articles with each section reviewed by two reviewers; those with two “yes” votes were moved forward to data extraction. The total number of records screened, reviewed, and remaining was recorded and presented in a figure prepared in accordance with the PRISMA-ScR statement [15].

### Data charting process

Two members of the team (M.M.N. and P.G.) pilot-tested an initial data extraction tool created by J.A. with two articles, one quantitative and one qualitative. This extraction tool was based in Covidence and contained both quantitative and qualitative data points clustered around study identifiers (year, geography, aims), cancer control elements (cancer type, location in cancer control continuum), and descriptions of champion elements (type, engagement style, title/role, training, implementation strategies, impact, facilitators/barriers, sustainability). Conflicts were resolved by discussing them with the entire group of authors and coming to consensus regarding the final format of the data extraction form. For data extraction, one of eight members of the research team (P.G., J.A., A.A., L.R.C., M.M.N., K.A.H., S.M.C., and A.M.L.-O.) independently extracted data from each article based on a predesigned extraction template using Covidence. Another member of this group completed a comparison of the extracted data with their examination of the article to confirm the extracted data were valid. If there were any conflicting decisions, the extraction datapoint was marked in Covidence “consensus required”, which were resolved by a third member of the team. No attempts were made to contact authors of the final articles, and only data in the data extraction report form were considered for the analysis.

The following data points were collected: (1) general study information, including last name of first author, title, year of publication, aim of the study, location, setting, type of community (i.e., urban, rural, suburban), study design, and methodological approach (i.e., quantitative, qualitative, mixed methods, community-based participatory); (2) characteristics of interventions and their participants, including stage in cancer care continuum (i.e., prevention, screening, treatment, survivorship/rehabilitation) and cancer type; (3) information on champions, including type of champions, type of engagement of champions, terms used when referring to champions, champion core activities (i.e., role in study team), how champions were trained and prepared for their role, complementary strategies (i.e., what other implementation strategies are bundled with champion approach), impact of the use of champions, and evaluation of champion experience; and (4) additional data on champions, including facilitators and barriers to using champions and the role of champions in sustainability of the intervention/program being evaluated.

### Thematic coding and synthesis of results

Characteristics of the included studies and data on the leveraging of champions were tabulated using the data extraction forms. Next, descriptive statistics were used to summarize core champion activities and complementary strategies. M.M.N. and J.A. then conducted a thematic analysis in order to develop a narrative synthesis. Specifically, they used both inductive and deductive coding approaches. Deductive codes were identified from the literature review. Inductive codes such as contextual facilitators and barriers to effective champion use, language used to describe champions, type of information collected regarding impact of champions, and the champion’s role in sustaining interventions or programs emerged. Then, both M.M.N. and J.A. initially coded the extracted data separately using Dedoose, a qualitative analysis program. They compared code applications and resolved conflicts through a consensus-building session. Codes were then refined in an iterative process based on the outcomes of these sessions. Lastly, M.M.N. built a model illustrating these codes emphasizing the roles, activities, and impacts of champions in cancer care initiatives.

## Results

### Selection of sources of evidence

**Fig. 1:**
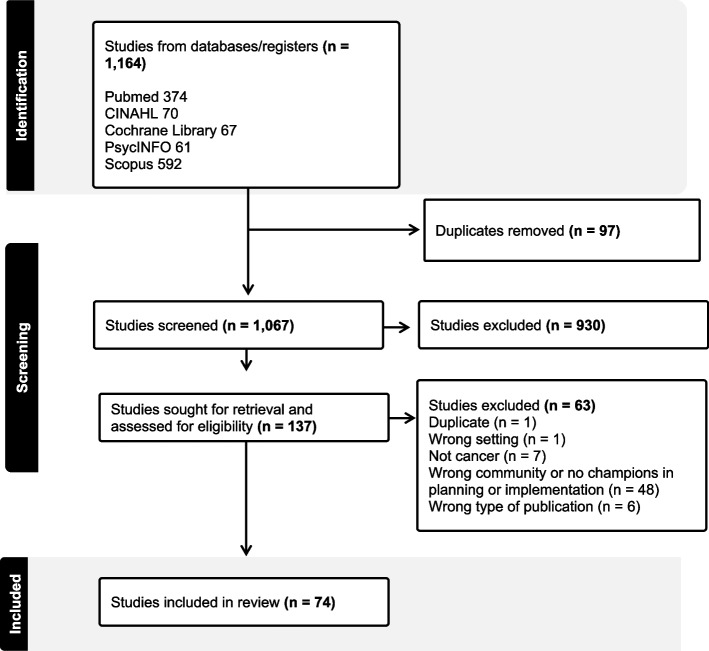
Prisma diagram of the study selection. Study identification, screening, and final inclusion in the scoping review are shown. Search strategies identified 1,164 citations total. Ninety-seven duplicates were removed, leaving a set of 1,067 citations for title/abstract screening. Of these, 137 articles were deemed relevant and 930 were excluded after initial screening by the team. The team then excluded 63 studies because 48 did not include community or clinical champions within translational research across the cancer care continuum (i.e., prevention, screening, treatment, survivorship/rehabilitation), 7 were not about cancer research, 6 were a type of publication not included in the review, and 1 was conducted in a setting not included in the review. A single duplicate study was also removed at this stage. A total of 74 articles were deemed eligible and included in the review

The search strategies identified 1,164 citations, which were exported to Covidence. Ninety-seven duplicates were removed, leaving a set of 1,067 citations for title/abstract screening. Of these, 137 articles were deemed relevant and were retrieved as full text. We excluded 63 studies because 48 did not include community or clinical champions as part of the translational research process within the cancer care continuum (i.e., prevention, screening, treatment, survivorship/rehabilitation), 7 were not about cancer research, 6 were a type of publication not included in the review such as conference proceedings, protocols, dissertations, or theses. One was conducted in a setting not included in the review (champions used in oncology care settings and community-based public health settings were the population of focus). A single duplicate study was also removed at this stage. These 63 studies are listed in Supplementary File 2. A total of 74 articles were deemed eligible and included in this review (see Supplementary File 3 for all included studies and their characteristics). The study selection process is shown in Fig. 1.

### Characteristics of studies included in the scoping review

Table 1 summarizes the characteristics of the 74 included studies. Research reports of champions increased between 2010 and 2020, and about 66% of the reviewed articles reported data collected in the United States. The included studies in this review utilized a range of research designs, providing a broad view of the evidence on the role of champions in cancer care implementation. A majority (33 studies) were descriptive, employing cross-sectional designs to explore various aspects of champion involvement and implementation outcomes. Eight studies used a comparative design, specifically randomized controlled trials (RCTs), to assess the effectiveness of champions by comparing intervention groups with control groups. Nineteen studies were non-comparative and observational in nature, focusing on the natural occurrence and influence of champions in real-world settings without control groups. Fourteen studies employed other designs, including case studies, quasi-experimental designs, and effectiveness implementation trials, contributing additional insights into the contexts and mechanisms through which champions facilitate change in healthcare settings. Most research was conducted in urban areas (57%), followed by rural (32%), and then suburban (22%). Champions appeared across interventions and/or programs addressing many cancer types (e.g., cervical, colorectal, breast, lung, skin, and prostate, among others). Champions were also used across the cancer care continuum, although they appeared most often in screening interventions (53%), and less frequently in prevention (27%), treatment (31%), and survivorship interventions (23%). Research on champions in intervention implementation was most often conducted in a medical center or hospital-based inpatient setting (41%), followed by a community-based setting (34%), and then a health clinic (19%). The status of the champions also varied along these lines; clinical champions were most common (61%), followed by community-based champions (22%), and then other champions (20%), leadership champions (16%), and finally patient champions (4%).

**Table 1:** Characteristics of the studies included in the scoping review (*n* = 74)

Variable	No. (%)
Year of study	
Before 2000	1 (1)
2000–2010	7 (9)
2011–2020	57 (77)
After 2020	5 (7)
Missing	4 (5)
Location	
Outside United States	24 (32)
United States	49 (66)
Missing	1 (1)
Geographic setting^a^	
Urban	42 (57)
Suburban	16 (22)
Rural	24 (32)
Missing	23 (31)
Institutional setting^a^	
Medical center or hospital-based inpatient	30 (41)
Community-based	25 (34)
Health clinics	14 (19)
Other	4 (5)
Missing	4 (5)
Location in cancer care continuum^a^	
Prevention	20 (27)
Screening	39 (53)
Treatment	23 (31)
Survivorship	17 (23)
Status of champion^a^	
Clinical	45 (61)
Community-based	16 (22)
Other	15 (20)
Leadership	12 (16)
Patient	3 (4)
Engagement of champion^a^	
Assigned	33 (45)
Volunteer	21 (28)
Emergent	13 (18)
Other	15 (20)
Missing	8 (11)
Contractor	1 (1)

^a^Categories were not mutually exclusive (some studies included more than one)

**Fig. 2:**
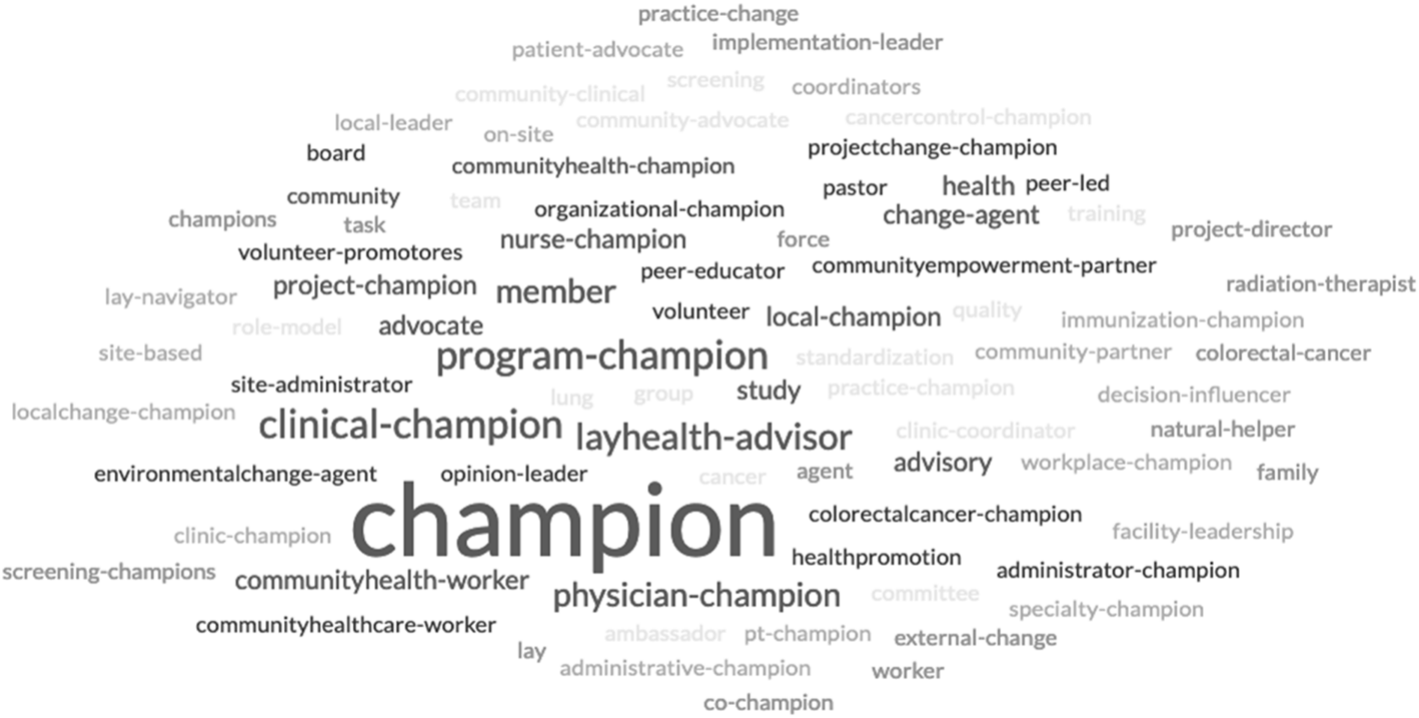
Word cloud of champion nomenclature. The word cloud was created by taking the free response data from the extraction form section asking what the champion was called in each of the 74 articles included in the scoping review. These data were then uploaded into a free word cloud generator. Free responses with multiple words were hyphenated to keep the words joined in the final figure

The current review also identified a variety of terms unrelated to the traditional “champion” nomenclature, including implementation leader, facilitator, liaison, promoter, opinion leader, decision influencer, lay health advisor, early adopter, and change agent, reflecting the multifaceted approaches to leadership and advocacy in cancer care (see Fig. 2). Cancer care champions were often from community-based contexts. These individuals served as lay health advisors, patient navigators, and/or community health workers, framing their vital work in cancer prevention for their peers and communities as the work of champions. Very few leadership or patient champions were reported in the results.

### Synthesis of results

#### Facilitators and barriers

Qualitative synthesis of the 74 included articles highlighted contextual elements that serve as either facilitators or barriers to the effective use of champions. The analysis revealed four primary facilitators for fostering an environment conducive to the emergence and success of champions. The first facilitator was paying attention to an individual’s characteristics (e.g., the champions’ personal attributes, skills, and experiences) that can play a pivotal role in their success. Champions who possess qualities such as intrinsic motivation [18], influence [19–21], and dedication [22] tend to excel in driving cancer care initiatives. The second theme was attention to role features; that is, the position and responsibilities assigned to a champion within an organization or initiative influence the champion’s reach and impact. These role features were broken down into financial incentives for community-based contexts [23], active support for champion activities within the role [24], and having champions from roles at multiple levels within an organization [25]. The third facilitator was organizational characteristics. Staff and leadership engagement with the intervention [26], communication style in the organization [27], and the training environment [27] in which the champion operates significantly affect the champion’s ability to operate successfully. Lastly, champions’ relationships with the target population were revealed to be important. That is, a strong link with the community can facilitate effective communication and enhance the champion’s credibility [28]. For example, involving the community in planning efforts for interventions allows scheduling of intervention components to be more intentional.

The review also identified several barriers that could hinder champions’ impact, including constrained time [24], low self-efficacy among champions [29], inadequate training [30], high turnover rates of champions [31], and a lack of buy-in from organizational leadership toward the intervention [32]. Data from the review showed that 45% of articles were not clear about whether the champions received any training.

#### Champion identification and core activities

Champions were commonly assigned their role; 45% of the included studies reported that champions were assigned by others in the organization. Volunteer champions (28%), followed by other (20%) and emergent champions (18%), were the next most frequently reported types of champions.

**Fig. 3:**
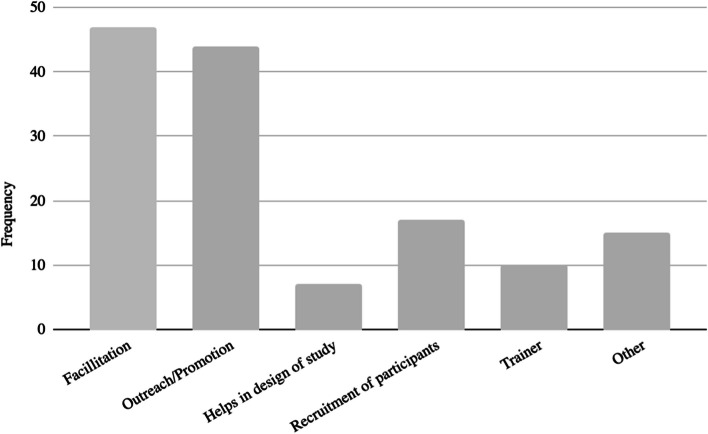
Champion core activities.This bar chart, created in Microsoft Excel, shows the six deductive categories describing champions’ core activities within implementation of cancer care interventions. Each of the articles within the scoping review was labeled with at least one of the tags. Percentages were calculated by dividing the total for each category by the total number of articles (74)

The most common clusters of champion activities reported included facilitation and outreach/promotion (Fig. 3). Less often, champions were involved in recruiting participants, training, and helping design the study. Within these broad clusters of activities, champions undertook several specific activities to drive initiatives forward. First, they were reported as assessing needs and opportunities; for example, one study involved champions in facilitation by asking them to identify gaps in care and potential areas for improvement in the implementation of the guidelines to improve constipation management in patients with cancer [33]. Next, champions were reported as working to empower the target population. Included studies mentioned that champions played a vital role in educating and enabling the target audience to take proactive measures in their care journey [34]. Champions often mentored and supported their peers. For example, one study used peer mentors within workplaces that had personal experiences with colon cancer [35]. Their role included implementing a communication plan with elements including social media messaging, guided conversations, and dissemination of information regarding training sessions [35]. Champions were also reported as engaging in and facilitating collaboration between different teams, departments, or even organizations to achieve collective goals [25]. For example, one study examining the facilitators and barriers to implementation of a mailed fecal immunochemical test identified having champions work as coordinators across executive and implementation levels of an intervention as integral to success [25]. Next, champions were reported as engaging in the identification and selection of other champions; champions understood the importance of continuity and identified and trained potential successors or additional champions, via the mechanism of behavioral modeling [21]. Lastly, champions often were described as responsible for monitoring the program or project [36]. For example, continuous assessment of the program’s effectiveness and making necessary adjustments were part and parcel of an HPV immunization champion’s role in monitoring performance data [36].

#### Complementary strategies

**Fig. 4:**
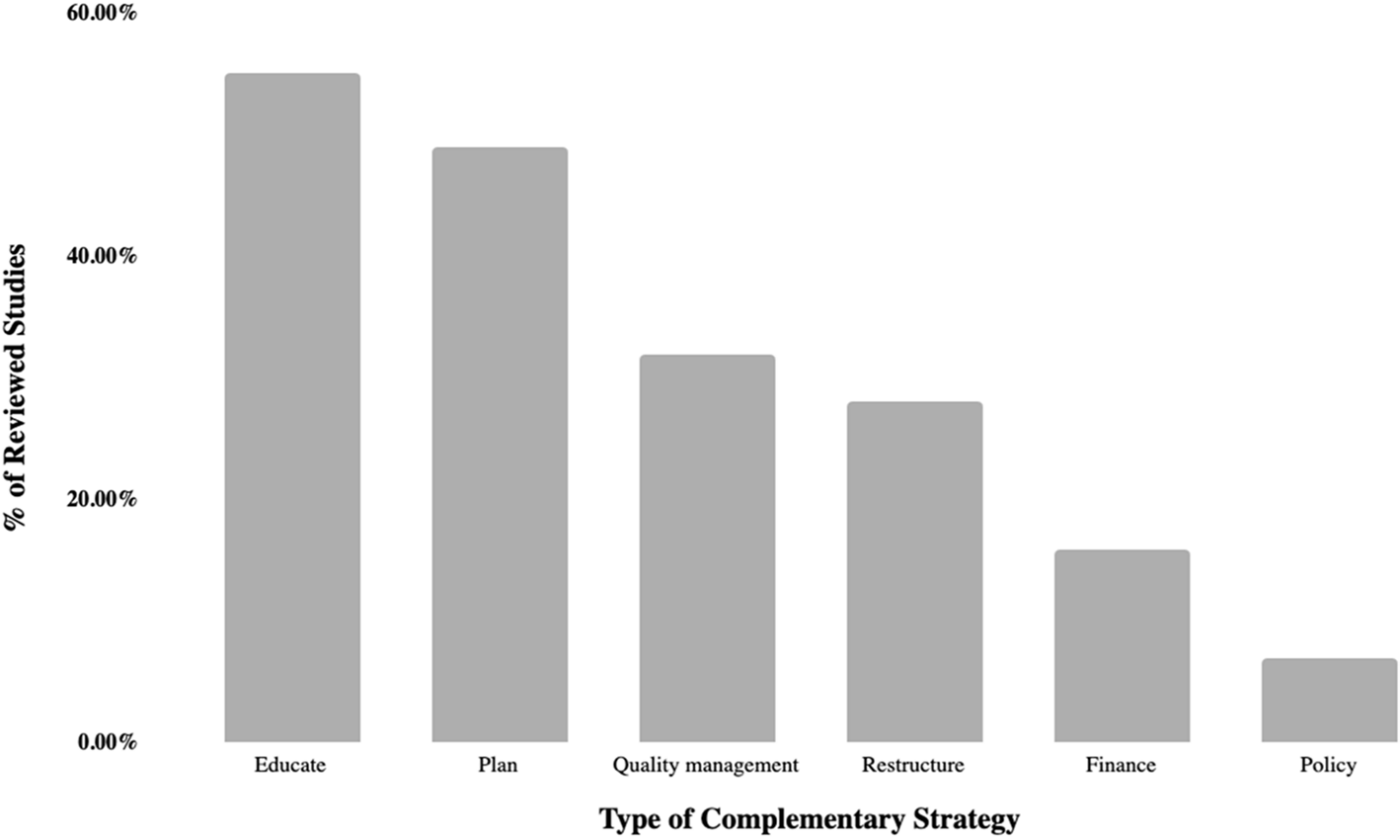
Complementary strategies. This bar chart, created in Microsoft Excel, shows the six deductive categories describing complementary strategies carried out alongside champion activation. Each of the articles within the scoping review was labeled with as many of these categories as identified. Percentages were calculated by dividing the total for each category by the total number of articles (74), and categories are shown from highest to lowest percentage

In many studies, the use of champions was described as a standalone implementation strategy. However, our review showed that champions also often complemented various activities within the implementation strategy, particularly planning and educational strategies (Fig. 4). Specific examples included community advisory board synergy with champions [37], train-the-trainer educational strategies alongside the presence of champions [26], using champions alongside audit and feedback processes as part of quality management [38], champion leadership in the evaluation of remunerative systems based on workload [39], and champion leveraging of federal funding to build leadership interest [40].

#### Champion impact and sustainability

Most studies (54%) did not have any data to illustrate the effect of the champion or evaluate the champion’s experience of the project (65%). Among the studies that reported on the effect of a champion (37%), evidence suggested that champion-driven initiatives can lead to several positive implementation outcomes. For example, our synthesis suggested that an environment that supports change [36] was cultivated when champions were included in the implementation process. Another observation was greater reach and clinical awareness when a project or initiative was integrated with champion strategies [22]. Some studies suggested that champions may reduce barriers to implementation by identifying the barriers and addressing these challenges [41]. Having a champion also helped to keep the initiative a priority despite other competing interests [42]. Lastly, using champions helped sustain a project beyond the initial implementation, allowing the effort to be continually maintained and improved upon [31], although most studies did not report or describe sustainability efforts (74%). Impact themes are summarized in Table 2.

**Table 2:** Impact themes

Theme	Reference
Developing a supporting climate for change	[22, 36, 41]
Greater reach and clinical awareness	[22, 27, 28, 30, 43, 45–52]
Reduction of barriers to implementation	[23, 41, 42, 45, 50]
Keeping the initiative a priority	[22, 41, 42, 53]
Sustainment of effort	[31, 41, 53]

#### The champion model

**Fig. 5:**
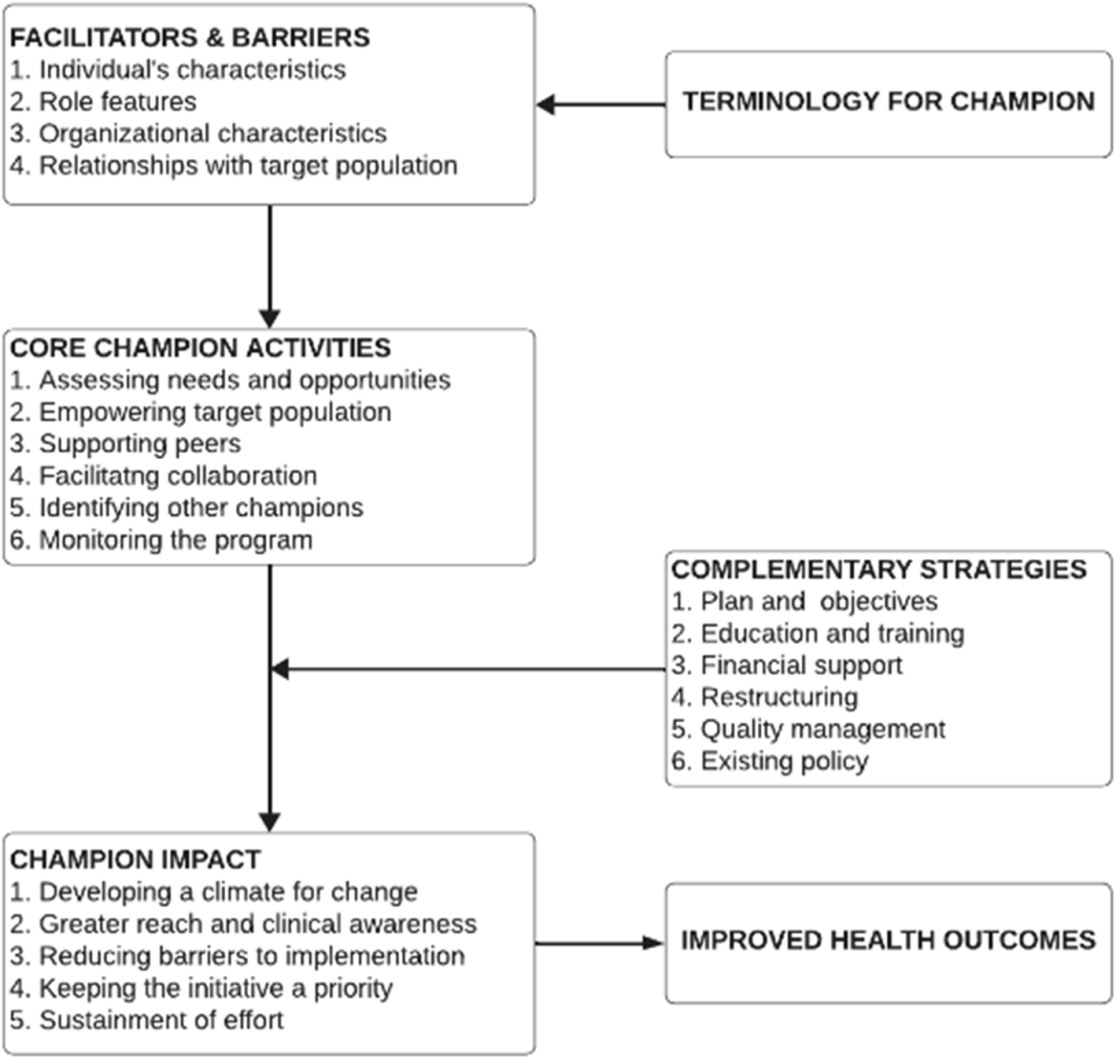
The Champion Model. The Champion Model is a conceptual framework developed by our team members, after they grouped information from the scoping review extraction form and discussed categories until consensus was reached. Specifically, free response data, including the words used to describe the champion, facilitators and barriers to use of champions, core activities, complementary strategies, and impact, were used to build the model. Health outcomes as a direct result of champions were not measured in any of the articles, but health outcomes are hypothesized to improve with the use of champions based on existing theoretical frameworks

Overall, our review provided comprehensive insights into the roles, activities, and impacts of champions in cancer care initiatives, which led us to develop a Champion Model (Fig. 5).

#### Terminology and role definition

Within the framework of the Champion Model, “champions” are identified as key personnel who advocate for and drive the implementation of health interventions. Their role is essential in catalyzing change, particularly in the domain of cancer care. The term “champion” conveys the function of individuals who leverage their expertise and influence to facilitate intervention uptake and integration into clinical and community settings.

#### Facilitators and barriers

Success in the implementation process is contingent upon the ability of champions to effectively manage facilitating factors and navigate barriers to implementation. The individual traits of a champion, such as credibility and commitment, can significantly influence their efficacy. The nature of the role a champion holds, coupled with the organizational context — including the institution’s capacity for change and available support systems — and the quality of the interaction with the target population, constitutes the environmental dynamics that either propel or impede progress.

#### Core activities of champions

The central tasks assigned to champions are critical to the momentum of the implementation process. These tasks range from assessing the landscape for needs and opportunities to actively monitoring the progress of the program. Champions are expected to engage in collaborative practices, promote peer support, and contribute to the strategic selection of new champions to build a resilient network that can sustain the intervention over time.

#### Complementary strategies

A series of supportive strategies is outlined to augment the efforts of champions. These strategies include planning to create a roadmap for implementation, educating to ensure all parties are informed, securing financing to sustain the intervention, restructuring as needed to accommodate the new practices, instituting quality management to uphold standards, and engaging in policy development to establish a supportive framework for the interventions.

#### Champion impact and health outcomes

The measurement of implementation impact is multi-dimensional, focusing on the ability to create an environment conducive to change, maintaining the initiative’s visibility and perceived importance, and ensuring that ongoing efforts do not wane. Champions are instrumental in maintaining a clear focus on these aspects to keep the initiative on course. The culmination of the Champion Model is observed in its impact on health outcomes within cancer care. The model posits that through strategic facilitation by champions, and by overcoming barriers to implementation, the desired health outcomes — notably in cancer prevention, treatment, and care — can be realized. This approach prioritizes a pragmatic and sustained application of evidence-based interventions to meet the complex challenges of cancer care delivery.

## Discussion

The results of the current scoping review indicated that the use of champions in translational cancer research increased between 2010 and 2020, and that most studies were conducted in urban areas of the United States. Champions as a component of translational research can be found in many cancer types and across the entire cancer care continuum. Despite a variety of terms being used to describe champions, clinical and community champions emerged as the most common. These champions are often assigned the role, and once assigned, champions are traditionally responsible for one or more of the following clusters of activities: (1) facilitation, (2) outreach/promotion, and (3) recruitment of participants. Less often, champions assisted with on-the-ground training and/or design of the study from its inception. Champions were also highly engaged with other types of implementation strategies ranging from planning to education, as well as quality management and policy/financial changes. This ranged from intentional community advisory board synergy with champions, the braiding of train-the-trainer educational strategies reinforced with champions, to the presence of champions working in parallel with the leveraging of new funding to attract leadership support. All of these examples demonstrate the value of champions as critical to generating momentum throughout the implementation process.

Despite their critical role, evidence about the impact of champions, specifically on health outcomes, is lacking. Around one-third of the studies in the current review mentioned some impacts in either qualitative or narrative format, specifically the effect of champions on creating and sustaining a climate for change.

Findings from our review uncovered that champions are often appointed to their roles based on their social position, rather than organizations identifying and activating emergent champions. The process of identifying emergent champions is not well understood, which may lead towards appointing champions as a standard practice. Position in an organization is often the criterion for selecting or appointing champions (clinical, community-based, leadership, or patient champions). Most research has framed champions as coming from clinical contexts [7], mirroring the results of the current review, which identified clinical champions in 61% of the studies. “Clinical” is a prevalent descriptor for champions, underscoring their authoritative role and expertise. These individuals are expected to use their position and expert knowledge to assist with implementation of a health initiative. The current scoping review also revealed a broadening of the use of champions; 22% of the studies contained a community-based champion. Surprisingly, leadership champions were found in only 16% of the articles, and patients in even fewer (4%). Future work examining best practices for leaders and patients enacting the role of a champion is needed.

Incentivizing champions is complex because traditionally both appointed and emergent champions are also not expected to receive any formal recognition or compensation [6]. Emergent champions are often seen as acting through charismatic leadership, rather than organizational norms and roles. Emergent champions are often described with personal attributes such as intrinsic motivation, commitment, ownership of the initiative, enthusiasm, persuasiveness, credibility, trustworthiness, holding influence through informal networks, physical presence, capacity to take on a new project, participatory leadership, and institutional savviness. All of these attributes may lead to assumptions that emergent champions transcend the need for traditional compensation and recognition. Simultaneously, little is known about the motivations of appointed champions. We do know that having an appointed champion without personal attributes such as ownership can prevent other champions from naturally emerging during the implementation [12] potentially causing a downward spiral of disengagement. One drawback to selecting champions based on personal characteristics is irreplaceability if a champion change occurs midway through implementation due to staff turnover, which has generally been found in public health [13]. Our review highlighted that while appointed champions bring structure and predictability, emergent champions are often valued for their ability to inspire and drive change organically. This duality suggests that a combination of appointed and emergent champions may be necessary for successful implementation.

Approaching champions with this duality of either appointed or emergent leads to social position or personal attributes embodying what a champion *is* rather than what they need to be able to do or what skills they possess to assist with implementation. This explains the lack of a common nomenclature and training documented in the literature. If a champion is defined by their position or personal characteristics, there is nothing that they need to learn to be a champion for an intervention. Trends were also uncovered when analyzing what cancer care champions are commonly asked to do to successfully implement interventions. In terms of core activities, champions play a pivotal role in not only assessing immediate needs but also empowering the individuals they serve. An emphasis on collaboration, through supporting peers or through inter-departmental or inter-organizational collaborations, is crucial for the successful implementation of cancer care programs. A noteworthy observation is the self-sustaining nature of champion-driven initiatives, as evident from the activity of identifying and training potential successors or additional champions, ensuring the continuity of the program’s efforts. These individuals often also do the emotional labor of overcoming resistance or pushback that comes with change management [8]. Skills to perform these tasks are not in-born in charismatic individuals or guaranteed due to the position one has in the health services system.

The components of the intervention also matter when planning for champions. For example, previous studies have revealed that one well-placed champion could implement a new technology [6]. However, more than one champion was needed when an improvement required people to change behaviors [6]. Although behavioral change itself may appear to be an inexpensive and simple solution, implementation is often more complicated than changing technology, because behavioral changes require interprofessional coalitions to work together [6]. Our review identified five articles that raised questions about the impact of a solo champion, finding that multiple champions had to work simultaneously in a coordinated way at a single site for successful implementation to occur [19, 25, 42–44]. In some cases, multiple champions were needed to perform study activities, and in others, a single individual was responsible for many aspects of the implementation process. Our findings also illustrate that champions often play a role in monitoring the program and sustaining its impact over time. Champions work to find other champions to replace themselves, as well as work to keep the initiative an organizational priority.

The current review has some limitations. The interplay between various contextual factors, core activities, and complementary strategies may vary depending on the specific type of cancer care initiative, making it essential to adapt and tailor approaches to the unique circumstances of each initiative. The role of a “champion” has also been named differently across studies. In the literature search, the most common names for this role were used; however, there may have been studies that used this role with a unique name that we did not identify. Future implementation research that uses a champion role should aim for a common nomenclature. 20% of the studies also did not identify the status of the champions (clinical/ community-based/ leadership/ patient), which should be considered when interpreting these results.

Researchers attempting to use champions are likely challenged by a lack of clarity in any single article reviewed. What is evident is that *championing* becomes a team sport. By intentional and in-depth focus on the “who” of implementation, interventionists can provide a more robust guide for replication. Champions need to be brought out from the background context of an organization, so that their work can be reported as part of the intervention and planned for future interventions proactively. For example, the Consolidated Framework for Implementation Research traditionally categorizes champions as part of the “engaging” construct even though the current review showed that champions are involved in many more activities than engagement alone [9]. Champions are also traditionally listed as only one standalone strategy in the Powell model of implementation strategies, with “identify and prepare champions” as a discrete planning strategy [8]. A new model is needed to underscore the importance of champions as part of the implementation context and as integrated with many standardized implementation strategies. The current scoping review may serve as a reference to assist organizations in identifying and activating effective champions for more specific contexts and interventions.

The recruitment and training of champions is not currently a standardized process. Despite the critical role of champions in ensuring the success of implementation, no studies in the review involved the development or validation of a standardized instrument that could differentiate among champion types, identify appropriate champions to use in interventions, or measure champion effectiveness. The current review illustrates that it is difficult to engineer the assigning of the champion role, making this process difficult to replicate across different contexts. The literature revealed a complex interplay of factors that contribute to the impact of champions in cancer care initiatives. This split between using appointed champions and volunteer/emergent champions can create tension in the field of implementation science, making it difficult to include “champions” as a discrete strategy in reporting on the intervention. Leaders may need to learn what interventions people are already champions for, align interventions with organizational goals, and elevate individuals to scale up their initial influence. These challenges underscore the complexity of implementing champion-led initiatives and highlight the need for comprehensive support and strategic planning to overcome these obstacles.

Standards for reporting in implementation science could also be clarified to allow for an operational definition of individuals deemed champions and their specific activities. This would allow for ease of replication and more generalizable knowledge about the process of identifying and activating the “right” champions for a specific cancer care intervention. Each cluster of activities identified in the current review could be used as a criterion to select appropriate champions depending on the needs of a specific intervention. Emergent champions could be assessed for their ability to perform the activities needed in the implementation process and then trained to complete these activities if they are not currently proficient. Both of these approaches would move beyond champions being defined by their status or personal attributes alone.

This review underscores the foundational role of champions in cancer care settings, whether in clinical environments (e.g., community health centers, academic medical centers) or community-based organizations. To effectively identify and activate champions, organizations should first determine whether they are aiming to identify emergent champions or appoint individuals to the role. Key characteristics such as intrinsic motivation, relevant experience, and strong community ties should be considered. In clinical settings, this involves selecting champions who are experienced enough to be considered influential and are also supported by the organization, while in community settings, it means finding individuals personally connected to and trusted by the community. Additionally, organizations should plan in advance the specific domains of tasks that champions will perform and identify which implementation strategies can be optimized once champions are activated. These results also suggest to first assess the level of buy-in from higher levels of the organizational hierarchy before beginning the formal champion activation process. By leveraging the insights from our review, organizations can more effectively match champions with implementation outcomes and optimize the overall impact of a variety cancer care interventions.

Future mixed methods research should delve deeper into understanding the nuances of champion-driven approaches across diverse settings, aiming to optimize the impact of champions in the ever-evolving landscape of cancer care. Case studies and/or qualitative comparative analysis could also unpack how complementary strategies depend on the use of champions and the degree to which this approach is correlated with effectiveness.

## Conclusions

The current scoping review may serve as a reference to assist organizations in identifying and activating effective champions for more specific contexts and interventions and help shape a nuanced approach to leveraging champions in the fight against cancer. Practitioners and policymakers within these organizations may consider champions as central figures in successfully implementing a range of cancer care initiatives across the cancer control continuum. When planning specific tasks for champions, practitioners should see their role as maximized when nested within a supportive environment that acknowledges and addresses the multifaceted nature of implementation challenges. In addition to champions being involved across the translational research process, they are also a cornerstone to other complementary implementation strategies. These strategies range from more immediate, actionable steps such as program planning and education to broader, systemic changes such as financing models, restructuring, quality management, and policy revisions. This reinforces the idea that champions are catalysts for change, but their impact is amplified when supported by comprehensive strategies that address the system at multiple levels. Champions play a critical role in ensuring the success of implementation by creating a climate for change, mitigating barriers to implementation, and increasing the intervention’s reach. Therefore, practitioners faced with barriers such as lack of readiness and limited scalability may consider first slowing down the implementation timeline and identifying emergent champions to increase overall impact. A champion’s work may be a key to optimizing overall health outcomes when activated through this pathway.

## Supplementary Information

Supplementary Material 1.

Supplementary Material 2.

Supplementary Material 3.

Supplementary Material 4.

## Data Availability

All data generated or analyzed during this study are included in this published article and its supplementary information files.
