# Supplementary material for: Champions to enhance implementation of clinical and community-based interventions in cancer: a scoping review
Source: Implement Sci Commun. 2024 Oct 22;5:119. doi: 10.1186/s43058-024-00662-0 (PMC11494796; doi:10.1186/s43058-024-00662-0)
Supplement: Supplementary file 1 — Supplementary Material 1. [file 43058_2024_662_MOESM1_ESM.docx]

**Supplementary File 1: Search Strategies**

PubMed (374)

(champion OR "change agent" OR "opinion leader" OR advoca*) AND (implementation OR "Implementation Science"[Mesh]) AND ("Neoplasms/prevention and control"[Mesh] OR "Neoplasms/therapy"[Mesh] OR "Cancer Survivors"[Mesh] OR "Public Health Practice"[Mesh] OR "Early Detection of Cancer"[Mesh] OR "Risk Reduction Behavior"[Mesh] OR "Adaptation, Psychological"[Mesh]) AND cancer

CINAHL (70)

(champion OR "change agent" OR "opinion leader" OR advoca*) AND (implementation OR MH "Implementation Science") AND ("cancer therapy" OR "Cancer prevention" OR MH "Cancer Survivors" OR "Public Health Practice" OR MH "Early Detection of Cancer" OR "Risk Reduction Behavior" OR MH "Adaptation, Psychological") AND (Cancer OR MH "Neoplasms")

Cochrane Library (44 trial records, 23 reviews = 67)

(champion OR "change agent" OR "opinion leader") AND cancer AND (prevention OR survivor OR risk)

PsycINFO (61)

(champion OR "change agent" OR "opinion leader" OR DE "Advocacy") AND (implementation OR DE "Quality of Care" OR DE "clinical practice" OR DE "quality control" OR DE "intervention" OR DE "Survivors" OR DE "Risk Management" OR DE "Health Risk Behavior") AND (DE "Neoplasms")

Scopus Advanced Search (592)

(champion OR "change agent" OR "opinion leader") AND (implementation OR INDEXTERMS("Implementation Science")) AND (INDEXTERMS("Neoplasms/prevention and control") OR INDEXTERMS(Neoplasms/therapy) OR INDEXTERMS("Cancer Survivors") OR INDEXTERMS("Public Health Practice") OR INDEXTERMS("Early Detection of Cancer") OR INDEXTERMS("Risk Reduction Behavior") OR INDEXTERMS("Adaptation, Psychological")) AND cancer
