# Supplementary material for: Champions to enhance implementation of clinical and community-based interventions in cancer: a scoping review
Source: Implement Sci Commun. 2024 Oct 22;5:119. doi: 10.1186/s43058-024-00662-0 (PMC11494796; doi:10.1186/s43058-024-00662-0)
Supplement: Supplementary file 2 — Supplementary Material 2. [file 43058_2024_662_MOESM2_ESM.docx]

**Supplementary File 2: Excluded Citations**

1. Abuadas FH, Petro-Nustas WJ, Abuadas MH. The effect of a health education intervention on Jordanian participants’ colorectal cancer knowledge, health perceptions, and screening practices. *Cancer Nursing*. 2018;41(3):226-237. doi:10.1097/NCC.0000000000000480

2. Afifi R, Person B, Haddad R. The impact of surgeons: pathologists dialog on lymph node evaluation of colorectal cancer patients. *Isr Med Assoc J*. 2018;20(1):30-33.

3. Ambler N, Rumsey N, Harcourt D, Khan F, Cawthorn S, Barker J. Specialist nurse counsellor interventions at the time of diagnosis of breast cancer: comparing “advocacy” with a conventional approach. *J Adv Nurs*. 1999;29(2):445-453. doi:10.1046/j.1365-2648.1999.00902.x

4. Anderson L, Adeney K, Shinn C, Safranek S, Buckner‐Brown J, Krause L. Community coalition‐driven interventions to reduce health disparities among racial and ethnic minority populations. *Cochrane Database Syst Rev*. 2015;6. doi:10.1002/14651858.CD009905.pub2

5. Andrews C, Childers TC, Wiseman KD, et al. Facilitators and barriers to reducing chemotherapy for early-stage breast cancer: a qualitative analysis of interviews with patients and patient advocates. *BMC Cancer*. 2022;22(1):141. doi:10.1186/s12885-022-09189-w

6. Bakitas M, Watts KA, Malone E, et al. Forging a new frontier: Providing palliative care to people with cancer in rural and remote areas. *J Clin Oncol*. 2020;38(9):963-973. doi:10.1200/JCO.18.02432

7. Chen F, Mercado C, Yermilov I, et al. Improving breast cancer quality of care with the use of patient navigators. *Am Surg*. 2010;76(10):1043-1046.

8. Clarke AL, Roscoe J, Appleton R, Dale J, Nanton V. “My gut feeling is we could do more...” a qualitative study exploring staff and patient perspectives before and after the implementation of an online prostate cancer-specific holistic needs assessment. *BMC Health Serv Res*. 2019;19(1):115. doi:10.1186/s12913-019-3941-4

9. Crevenna R, Kainberger F, Wiltschke C, et al. Cancer rehabilitation: current trends and practices within an Austrian University Hospital Center*. *Disabil Rehabil*. 2020;42(1):2-7. doi:10.1080/09638288.2018.1514665

10. Davis C, Darby K, Moore M, Cadet T, Brown G. Breast care screening for underserved African American women: Community-based participatory approach. *J Psychosoc Oncol*. 2017;35(1):90-105. doi:10.1080/07347332.2016.1217965

11. de la Riva EE, Hajjar N, Tom LS, Phillips S, Dong X, Simon MA. Providers’ views on a community-wide patient navigation program: Implications for dissemination and future implementation. *Health Promot Pract*. 2016;17(3):382-390. doi:10.1177/1524839916628865

12. Deverka PA, Bangs R, Kreizenbeck K, et al. A new framework for patient engagement in cancer clinical trials cooperative group studies. *J Natl Cancer Inst*. 2018;110(6):553-559. doi:10.1093/jnci/djy064

13. Ehlers SL, Davis K, Bluethmann SM, et al. Screening for psychosocial distress among patients with cancer: implications for clinical practice, healthcare policy, and dissemination to enhance cancer survivorship. *Transl Behav Med*. 2019;9(2):282-291. doi:10.1093/tbm/iby123

14. Engelman KK, Cupertino AP, Daley CM, et al. Engaging diverse underserved communities to bridge the mammography divide. *BMC Public Health*. 2011;11. doi:10.1186/1471-2458-11-47

15. Ferrante JM, Chen PH, Kim S. The effect of patient navigation on time to diagnosis, anxiety, and satisfaction in urban minority women with abnormal mammograms: a randomized controlled trial. *J Urban Health*. 2008;85(1):114-124. doi:10.1007/s11524-007-9228-9

16. Garland SN, Trevino K, Liou KT, et al. Multi-stakeholder perspectives on managing insomnia in cancer survivors: recommendations to reduce barriers and translate patient-centered research into practice. *J Cancer Surviv*. 2021;15(6):951-960. doi:10.1007/s11764-021-01001-1

17. Ghaffari M, Esfahani S, Rakhshanderou S, Koukamari P. Evaluation of health belief model-based intervention on breast cancer screening behaviors among health volunteers. 2019;34(5):904‐912. doi:10.1007/s13187-018-1394-9

18. Hedlund S. Northwest cancer specialists (ncs) cares: coordinated, advocacy, resources, education, and support: a palliative care program in an outpatient oncology practice. *Omega (Westport)*. 2013;67(1-2):109-113. doi:10.2190/OM.67.1-2.l

19. Howard AF, Smillie K, Chan V, Cook S, Kazanjian A. The knowledge exchange-decision support model: Application to cancer navigation programs. *Support Care Cancer*. 2014;22(2):367-374. doi:10.1007/s00520-013-1982-5

20. Hubbard G, Kidd L, Donaghy E, McDonald C, Kearney N. A review of literature about involving people affected by cancer in research, policy and planning and practice. *Patient Educ Couns*. 2007;65(1):21-33. doi:10.1016/j.pec.2006.02.009

21. Inzetta S. The breast imaging nurse navigator: Measuring the impact on coordinated care delivery - results after the first year. Academy of Oncology Nurse & Patient Navigators, November 17-20, 2016, Las Vegas, Nevada. *J Oncol Navig Surviv*. 2016;7(9):36-36.

22. Jones M, Ross B, Cloth A, Heller L. Interventions to reach underscreened populations: a narrative review for planning cancer screening initiatives. *Int J Public Health*. 2015;60(4):437-447. doi:10.1007/s00038-015-0666-y

23. Keehn DC, Chamberlain RM, Tibbits M, Kahesa C, Msami K, Soliman AS. Using key informants to evaluate barriers to education and acceptability of the HPV vaccine in Tanzania: Implications for cancer education. *J Cancer Educ*. 2021;36(6):1333-1340. doi:10.1007/s13187-020-01773-7

24. Kumar A, Nesbitt KM, Bakkum-Gamez JN. Quality improvement in gynecologic oncology: Current successes and future promise. *Gynecol Oncol*. 2019;152(3):486-491. doi:10.1016/j.ygyno.2018.10.046

25. Laurent-Ledru V, Thomson A, Monsonego J. Civil society: A critical new advocate for vaccination in Europe. *Vaccine*. 2011;29(4):624-628. doi:10.1016/j.vaccine.2010.11.004

26. LeClair AM, Battaglia TA, Casanova NL, et al. Assessment of patient navigation programs for breast cancer patients across the city of Boston. *Support Care Cancer*. 2022;30(3):2435-2443. doi:10.1007/s00520-021-06675-y

27. Liu D, Schuchard H, Burston B, Yamashita T, Albert S. Interventions to reduce healthcare disparities in cancer screening among minority adults: A systematic review. *J Racial Ethn Health Disparities*. 2021;8(1):107-126. doi:10.1007/s40615-020-00763-1

28. Lockwood-Rayermann S, McIntyre SJ. Understanding HPV disease and prevention: A guide for school nurses. *J Sch Nurs*. 2009;25(4):261-269. doi:10.1177/1059840509333787

29. Lynge E, Törnberg S, von Karsa L, Segnan N, van Delden JJ. Determinants of successful implementation of population-based cancer screening programmes. *Eur J Cancer*. 2012;48(5):743-748. doi:10.1016/j.ejca.2011.06.051

30. McCusker J, Yaffe M, Faria R, et al. Phase II trial of a depression self-care intervention for adult cancer survivors. *Eur J Cancer Care*. 2018;27(1). doi:10.1111/ecc.12763

31. McWilliams L, Bellhouse S, Yorke J, Cowan R, Heaven CM, French DP. The acceptability and feasibility of lay-health led interventions for the prevention and early detection of cancer. *Psycho-Oncology*. 2018;27(4):1291-1297. doi:10.1002/pon.4670

32. Mojica CM, Morales-Campos DY, Carmona CM, Ouyang Y, Liang Y. Breast, cervical, and colorectal cancer education and navigation: Results of a community health worker intervention. *Health Promot Pract*. 2016;17(3):353-363. doi:10.1177/1524839915603362

33. Müller E, Hahlweg P, Scholl I. What do stakeholders need to implement shared decision making in routine cancer care? A qualitative needs assessment. *Acta Oncol*. 2016;55(12):1484-1491. doi:10.1080/0284186X.2016.1227087

34. Nawwar A, Brand Bateman L, Khamess S, et al. Using intervention mapping to develop a theory-based intervention to promote colorectal cancer screening in Egypt. *Asian Pac J Cancer Prev*. 2022;23(6):1975-1981. doi:10.31557/APJCP.2022.23.6.1975

35. NCT02067507. Increasing human papillomavirus vaccine uptake in low-income, ethnic minority adolescents in Los Angeles County. Published online 2014. https://www.cochranelibrary.com/central/doi/10.1002/central/CN-01543818/full

36. NCT04446728. Implementation of family psychosocial risk assessment in pediatric cancer. Published online 2020. https://www.cochranelibrary.com/central/doi/10.1002/central/CN-02133908/full

37. Parker BW, McAneny BL, Mitchell EP, et al. Establishing a primary care alliance for conducting cancer prevention clinical research at community sites. *Cancer Prev Res (Phila)*. 2021;14(11):977-982. doi:10.1158/1940-6207.CAPR-21-0019

38. Pfeifer MP, Ritchie C, Scharfenberger J, et al. The Caring Connections Project: Providing palliative care to Medicaid patients with advanced cancer. *Lippincotts Case Manag*. 2006;11(6):318-326. doi:10.1097/00129234-200611000-00007

39. Phillips-Angeles E, Song L, Hannon PA, et al. Fostering partnerships and program success. *Cancer*. 2013;119(suppl.15):2884-2893. doi:10.1002/cncr.28157

40. Pinder LF, Henry-Tillman R, Linyama D, et al. Leverage of an existing cervical cancer prevention service platform to initiate breast cancer control services in Zambia: experiences and early outcomes. *J Glob Oncol*. 2018;4:1-8. doi:10.1200/JGO.17.00026

41. Platner JH, Bennett LM, Millikan R, Barker MD. The partnership between breast cancer advocates and scientists. *Environ Mol Mutagen*. 2002;39(2-3):102-107. doi:10.1002/em.10055

42. Puleo E, Zapka J, White MJ, Mouchawar J, Somkin C, Taplin S. Caffeine, cajoling, and other strategies to maximize clinician survey response rates. *Eval Health Prof*. 2002;25(2):169-184. doi:10.1177/016327870202500203

43. Riogi B, Ross C, Mutebi M, Dave RV. The Kenya UK Breast Cancer Awareness Week: curriculum codesign and codelivery with direct and lived experience of breast cancer diagnosis and management. *BMJ Glob Health*. 2022;7(5). doi:10.1136/bmjgh-2022-008755

44. Rose SB, Lanumata T, Lawton BA. Promoting uptake of the HPV vaccine: the knowledge and views of school staff. *J Sch Health*. 2011;81(11):680-687. doi:10.1111/j.1746-1561.2011.00644.x

45. Roy S, Dickey S, Wang HL, et al. Systematic review of interventions to increase stool blood colorectal cancer screening in African Americans. *J Community Health*. 2021;46(1):232-244. doi:10.1007/s10900-020-00867-z

46. Russell K, Champion V, Monahan P, et al. Randomized trial of a lay health advisor and computer intervention to increase mammography screening in African American women. 2010;19(1):201‐210. doi:10.1158/1055-9965.EPI-09-0569

47. Sandiford L, D’Errico EM. Facilitating shared decision making about prostate cancer screening among African American men. *Oncol Nurs Forum*. 2016;43(1):86-92. doi:10.1188/16.ONF.86-92

48. Sandoval C, Cáceres CF. Influence of health rights discourses and community organizing on equitable access to health: the case of HIV, tuberculosis and cancer in Peru. *Global Health*. 2013;9:23. doi:10.1186/1744-8603-9-23

49. Smits S, McCutchan G, Wood F, et al. Development of a behavior change intervention to encourage timely cancer symptom presentation among people living in deprived communities using the behavior change wheel. *Ann Behav Med*. 2018;52(6):474-488. doi:10.1007/s12160-016-9849-x

50. Somayaji D, Blok AC, Hayman LL, Colson Y, Jaklisch M, Cooley ME. Enhancing behavioral change among lung cancer survivors participating in a lifestyle risk reduction intervention: a qualitative study. *Supportive Care Cancer*. 2019;27(4):1299-1308. doi:10.1007/s00520-018-4631-1

51. Taylor C, Cummings R, McGilly C. Holistic needs assessment following colorectal cancer treatment. *Gastrointestinal Nursing*. 2012;10(9):42-49. doi:10.12968/gasn.2012.10.9.42

52. Thorne S, Truant T. Will designated patient navigators fix the problem? Oncology nursing in transition. *Can Oncol Nurs J*. 2010;20(3):116-128. doi:10.5737/1181912x203116121

53. Truccolo I, Cipolat Mis C, Cervo S, et al. Patient-centered cancer care orograms in Italy: Benchmarking global patient education initiatives. *J Cancer Educ*. 2016;31(2):405-412. doi:10.1007/s13187-015-0805-4

54. Tsu VD, Pollack AE. Preventing cervical cancer in low-resource settings: how far have we come and what does the future hold? *Int J Gynaecol Obstet*. 2005;89(Suppl.2):S55-9. doi:10.1016/j.ijgo.2005.01.011

55. Turner J, Mackenzie L, Kelly B, Clarke D, Yates P, Aranda S. Building psychosocial capacity through training of front-line health professionals to provide brief therapy: lessons learned from the PROMPT study. *Support Care Cancer*. 2018;26(4):1105-1112. doi:10.1007/s00520-017-3929-8

56. Vallet F, Guillaume E, Dejardin O, et al. Influence of a screening navigation program on social inequalities in health beliefs about colorectal cancer screening. *J Health Psychol*. 2016;21(8):1700-1710. doi:10.1177/1359105314564018

57. Vrdoljak E, Gligorov J, Wierinck L, et al. Addressing disparities and challenges in underserved patient populations with metastatic breast cancer in Europe. *Breast*. 2021;55:79-90. doi:10.1016/j.breast.2020.12.005

58. Weber JJ, Mascarenhas DC, Bellin LS, Raab RE, Wong JH. Patient navigation and the quality of breast cancer care: an analysis of the breast cancer care quality indicators. *Ann Surg Oncol*. 2012;19(10):3251-3256. doi:10.1245/s10434-012-2527-8

59. Wells KJ, Battaglia TA, Dudley DJ, et al. Patient navigation: State of the art or is it science? *Cancer*. 2008;113(8):1999-2010. doi:10.1002/cncr.23815

60. Yan AF, Stevens P, Holt C, et al. Culture, identity, strength and spirituality: A qualitative study to understand experiences of African American women breast cancer survivors and recommendations for intervention development. *Eur J Cancer Care*. 2019;28(3). doi:10.1111/ecc.13013

61. Yancey AK. The meta-volition model: Organizational leadership is the key ingredient in getting society moving, literally! *Prev Med*. 2009;49(4):342-351. doi:10.1016/j.ypmed.2009.09.004

62. Zebrack BJ, Oeffinger KC, Hou P, Kaplan S. Advocacy skills training for young adult cancer survivors: the Young Adult Survivors Conference at Camp Māk-a-Dream. *Support Care Cancer*. 2006;14(7):779-782. doi:10.1007/s00520-005-0906-4

63. Zoellner J, Porter K, Thatcher E, et al. A multilevel approach to understand the context and potential solutions for low colorectal cancer (CRC) screening rates in rural Appalachia clinics. *J Rural Health*. 2021;37(3):585-601. doi:10.1111/jrh.12522
