# Supplementary material for: Champions to enhance implementation of clinical and community-based interventions in cancer: a scoping review
Source: Implement Sci Commun. 2024 Oct 22;5:119. doi: 10.1186/s43058-024-00662-0 (PMC11494796; doi:10.1186/s43058-024-00662-0)
Supplement: Supplementary file 3 — Supplementary Material 3. [file 43058_2024_662_MOESM3_ESM.docx]

**Supplementary File 3: Included Study Characteristics and Citations**

| Authors | Geographic Setting | Institutional Setting | Study Type | Location in Cancer Care Continuum | Status of Champion | Engagement of Champion |
| --- | --- | --- | --- | --- | --- | --- |
| Adegboyega et al. (2021) | Rural | Medical Center or Hospital-based inpatient | Comparative (randomized control trial) | Screening | Other | Other |
| Allen et al. (2021) | Urban; Rural | Health Clinics | Non-comparative (observational) | Screening | Leadership; Other | Emergent; Other |
| Aoun et al. (2013) | Urban; Rural; Suburban | Community-based | Descriptive (cross-sectional) | Prevention | Community-based | Emergent; Volunteer |
| Behringer et al. (2010) | Urban; Rural; Suburban | Other | Descriptive (cross-sectional) | Prevention; Screening; Treatment; Survivorship/Rehab | Clinical; Community-based; Patient | Volunteer; Other |
| Beidas et al. (2014) |  | Medical Center or Hospital-based inpatient | Other: Effectiveness- implementation trial | Survivorship/Rehab | Clinical | Assigned |
| Benard et al. (2014) | Urban | Health Clinics | Comparative (randomized control trial) | Screening | Other | Assigned |
| Birken et al. (2018) |  | Medical Center or Hospital-based inpatient |  | Survivorship/Rehab | Leadership; Clinical | Assigned |
| Bowman et al. (2015) |  | Medical Center or Hospital-based inpatient | Descriptive (cross-sectional) | Screening; Treatment | Leadership | Volunteer; Other |
| Campbell et al. (2011) | Urban | Medical Center or Hospital-based inpatient | Descriptive (cross-sectional) | Survivorship/Rehab | Leadership; Clinical | Emergent |
| Chuang et al. (2017) |  | Health Clinics | Descriptive (cross-sectional) | Prevention | Clinical | Assigned |
| Currier et al. (2022) | Urban | Medical Center or Hospital-based inpatient | Descriptive (cross-sectional) | Screening | Leadership; Clinical | Volunteer; Assigned |
| DeGroff et al. (2019) |  | Community-based | Non-comparative (observational) | Screening | Leadership; Clinical | Volunteer; Assigned |
| Dudgeon et al. (2012) |  | Medical Center or Hospital-based inpatient | Non-comparative (observational) | Screening | Clinical | Volunteer |
| Duong et al. (2021) | Urban; Suburban | Community-based | Descriptive (cross-sectional) | Screening | Community-based | Other |
| Escoffery et al. (2019) | Urban; Rural; Suburban | Community-based | Descriptive (cross-sectional) | Prevention | Leadership; Clinical | Volunteer; Assigned |
| Friedman et al. (2009) |  | Community-based | Comparative (randomized control trial) | Treatment | Leadership; Clinical | Assigned |
| Friese et al. (2017) |  | Health Clinics | Descriptive (cross-sectional) | Prevention; Treatment; Survivorship/Rehab | Clinical | Assigned |
| Gesthalter et al. (2017) | Urban | Medical Center or Hospital-based inpatient | Descriptive (cross-sectional) | Screening | Leadership; Clinical | Volunteer; Assigned |
| Gilleland Marchak et al. (2021) | Urban | Medical Center or Hospital-based inpatient | Descriptive (cross-sectional) | Treatment | Clinical; Other | Emergent |
| Ginex et al. (2021) |  | Health Clinics | Non-comparative (observational) | Treatment | Clinical | Volunteer; Assigned |
| Glaser et al. (2019) | Urban | Medical Center or Hospital-based inpatient | Descriptive (cross-sectional) | Survivorship/Rehab | Clinical | Emergent; Volunteer |
| Gulasingam et al. (2019) | Urban | Medical Center or Hospital-based inpatient | Descriptive (cross-sectional) | Screening | Clinical | Assigned |
| Hämmerli et al. (2022) |  | Community-based | Descriptive (cross-sectional) | Screening | Community-based | Volunteer |
| Haugen et al. (2016) | Urban | Medical Center or Hospital-based inpatient | Descriptive (cross-sectional) | Treatment; Survivorship/Rehab | Clinical | Assigned |
| Hempstead et al. (2018) | Urban | Medical Center or Hospital-based inpatient | Descriptive (cross-sectional) | Prevention | Community-based | Volunteer |
| Hohl et al. (2022) | Urban; Rural | Medical Center or Hospital-based inpatient | Descriptive (cross-sectional) | Prevention; Treatment | Clinical | Emergent; Assigned |
| Hu et al. (2019) |  | Medical Center or Hospital-based inpatient | Other | Treatment | Clinical | Assigned |
| Iwata et al. (2019) | Urban | Medical Center or Hospital-based inpatient | Descriptive (cross-sectional) | Survivorship/Rehab | Clinical | Emergent |
| Kennedy et al. (2014) | Urban; Rural; Suburban | Medical Center or Hospital-based inpatient | Other | Treatment | Clinical | Emergent |
| Knerr et al. (2020) |  | Medical Center or Hospital-based inpatient; Health Clinics | Non-comparative (observational) | Prevention | Clinical; Other | Emergent; Assigned |
| Lee et al. (2019) |  | Community-based | Comparative (randomized control trial) | Screening | Community-based | Volunteer |
| Lee at al. (2017) | Rural | Health Clinics | Other | Screening | Clinical; Community-based; Other | Other |
| Leeman et al. (2020) |  | Health Clinics | Descriptive (cross-sectional) | Prevention; Screening | Other | Assigned |
| Loo et al. (2022) | Urban | Medical Center or Hospital-based inpatient | Descriptive (cross-sectional) | Treatment; Survivorship/Rehab | Clinical | Other |
| Lovell et al. (2022) | Urban; Suburban | Health Clinics | Comparative (randomized control trial) | Treatment; Survivorship/Rehab | Clinical |  |
| Mahloch et al. (1993) | Urban; Rural; Suburban | Health Clinics | Descriptive (cross-sectional) | Screening | Clinical | Assigned |
| McBride et al. (2018) | Rural | Community-based | Non-comparative (observational) | Screening | Clinical | Assigned |
| Mittal et al. (2022) |  | Medical Center or Hospital-based inpatient | Descriptive (cross-sectional) | Prevention; Screening; Treatment; Survivorship/Rehab | Clinical | Assigned |
| Molina et al. (2022) | Urban | Medical Center or Hospital-based inpatient | Comparative (randomized control trial) | Screening | Patient | Emergent |
| O’Donovan et al. (2021) |  |  | Descriptive (cross-sectional) | Screening | Clinical |  |
| Payne et al. (2022) | Urban | Community-based | Descriptive (cross-sectional) | Prevention; Screening | Community-based | Assigned |
| Phillips-Angeles et al. (2013) |  | Community-based | Descriptive (cross-sectional) | Screening | Clinical | Assigned |
| Rafie et al. (2020) |  | Community-based | Non-comparative (observational) | Prevention; Screening | Other | Volunteer |
| Rao et al. (2022) | Urban | Medical Center or Hospital-based inpatient | Non-comparative (observational) | Treatment | Clinical | Assigned |
| Redwood et al. (2011) | Urban | Community-based | Non-comparative (observational) | Screening | Clinical | Volunteer; Other |
| Rhode et al. (2020) |  | Community-based; Health Clinics | Comparative (randomized control trial) | Screening | Leadership; Other | Emergent; Assigned |
| Ribeiro et al. (2016) | Urban; Rural; Suburban | Other | Other | Treatment | Leadership; Clinical; Other | Other |
| Robinson et al. (2017) | Urban | Medical Center or Hospital-based inpatient | Other | Treatment | Clinical | Emergent |
| Rocque et al. (2016) | Urban; Rural; Suburban | Medical Center or Hospital-based inpatient; Other | Non-comparative (observational) | Treatment; Survivorship/Rehab | Leadership | Other |
| Rogers et al. (2019) | Rural | Community-based | Non-comparative (observational) | Survivorship/Rehab | Other | Other |
| Schoenberg et al. (2009) | Rural | Community-based | Other | Screening | Community-based | Assigned |
| Sendall et al. (2016) | Rural | Community-based | Non-comparative (observational) | Prevention | Community-based | Assigned |
| Sharma et al. (2021) | Urban; Rural | Community-based | Other | Screening | Other |  |
| Sharma et al. (2021) | Urban; Rural; Suburban | Health Clinics | Other | Screening |  |  |
| Shelton et al. (2017) | Urban | Community-based | Descriptive (cross-sectional) | Prevention; Screening | Community-based | Volunteer; Other |
| Simunovic et al. (2013) | Urban; Suburban | Medical Center or Hospital-based inpatient | Comparative (randomized control trial) | Treatment | Clinical | Assigned |
| Slatore et al. (2021) | Rural | Medical Center or Hospital-based inpatient | Descriptive (cross-sectional) | Screening | Clinical; Other | Emergent |
| Sopcak et al. (2016) | Urban; Rural | Health Clinics; Other |  | Prevention; Screening | Clinical | Assigned |
| Stacey et al. (2020) | Urban; Rural; Suburban | Medical Center or Hospital-based inpatient | Other | Treatment | Clinical |  |
| Steinberg et al. (2006) | Urban |  |  | Prevention | Community-based | Volunteer |
| Szeszulski et al. (2022) |  |  | Other | Screening | Clinical; Community-based |  |
| Thompson et al. (2006) | Rural | Community-based | Other: Cross-sectional | Prevention; Screening | Leadership; Other | Volunteer |
| Townsend et al. (2011) | Urban; Suburban | Community-based | Descriptive (cross-sectional) | Prevention |  |  |
| Truant et al. (2017) | Urban; Rural; Suburban | Other | Non-comparative (observational) | Treatment | Clinical | Assigned |
| Urquhart et al. (2019) |  |  | Non-comparative (observational) | Treatment; Survivorship/Rehab | Clinical |  |
| Urquhart et al. (2014) | Urban; Suburban | Medical Center or Hospital-based inpatient | Non-comparative (observational) | Screening; Treatment | Clinical | Assigned |
| Warner et al. (2015) | Urban | Medical Center or Hospital-based inpatient | Descriptive (cross-sectional) | Survivorship/Rehab | Clinical | Other |
| Watson et al. (2021) | Urban; Rural | Health Clinics | Other | Screening | Clinical; Other | Other |
| Williams et al. (2020) | Urban; Suburban | Community-based | Non-comparative (observational) | Prevention; Screening | Community-based; Other | Volunteer; Assigned |
| Williams et al. (2015) |  | Community-based; Medical Center or Hospital-based inpatient; Health Clinics | Descriptive (cross-sectional) | Survivorship/Rehab | Clinical | Assigned |
| Williams et al. (2019) | Urban | Community-based | Other | Prevention; Screening | Leadership; Community-based | Volunteer; Other |
| Williams et al. (2021) | Rural | Community-based | Non-comparative (observational) | Screening | Community-based | Contractor |
| Wright et al. (2019) | Urban | Medical Center or Hospital-based inpatient | Non-comparative (observational) | Treatment | Clinical | Assigned |
| Yan et al. (2022) |  | Community-based | Non-comparative (observational) | Screening | Patient | Volunteer |

1. Adegboyega A, Aleshire M, Wiggins AT, Palmer K, Hatcher J. A motivational interviewing intervention to promote CRC screening: A pilot study. *Cancer Nurs*. 2021;45(1):E229-E237. doi:10.1097/NCC.0000000000000905

2. Allen CG, Cotter MM, Smith RA, Watson L. Successes and challenges of implementing a lung cancer screening program in federally qualified health centers: a qualitative analysis using the Consolidated Framework for Implementation Research. *Transl Behav Med*. 2021;11(5):1088-1098. doi:10.1093/tbm/ibaa121

3. Aoun S, Shahid S, Le L, Holloway K. Champions in a lifestyle risk-modification program: reflections on their training and experiences. *Health Promot J Austr*. 2013;24(1):7-12. doi:10.1071/HE12904

4. Behringer B, Lofton S, Knight ML. Models for local implementation of comprehensive cancer control: Meeting local cancer control needs through community collaboration. *Cancer Causes Control*. 2010;21(12):1995-2004. doi:10.1007/s10552-010-9655-x

5. Beidas RS, Paciotti B, Barg F, et al. A hybrid effectiveness-implementation trial of an evidence-based exercise intervention for breast cancer survivors. *J Natl Cancer Inst Monogr*. 2014;2014(50):338-345. doi:10.1093/jncimonographs/lgu033

6. Benard V, Saraiya M, Greek A, et al. Overview of the CDC Cervical Cancer (Cx3) Study: An educational intervention of HPV testing for cervical cancer screening. 2014;23(3):197‐203. doi:10.1089/jwh.2013.4655

7. Birken SA, Clary AS, Bernstein S, et al. Strategies for successful survivorship care plan implementation: Results from a qualitative study. *J Oncol Pract*. 2018;14(8):e462-e483. doi:10.1200/JOP.17.00054

8. Bowman C, Luck J, Gale RC, Smith N, York LS, Asch S. A qualitative evaluation of web-based cancer care quality improvement toolkit use in the Veterans Health Administration. *Qual Manag Health Care*. 2015;24(3):147-161. doi:10.1097/QMH.0000000000000063

9. Campbell MK, Tessaro I, Gellin M, et al. Adult cancer survivorship care: Experiences from the LIVESTRONG centers of excellence network. *J Cancer Surv*. 2011;5(3):271-282. doi:10.1007/s11764-011-0180-z

10. Chuang E, Cabrera C, Mak S, Glenn B, Hochman M, Bastani R. Primary care team- and clinic level factors affecting HPV vaccine uptake. *Vaccine*. 2017;35(35 Pt B):4540-4547. doi:10.1016/j.vaccine.2017.07.028

11. Currier J, Howes D, Cox C, et al. A coordinated approach to implementing low-dose CT lung cancer screening in a rural community hospital. *J Am Coll Radiol*. 2022;19(6):757-768. doi:10.1016/j.jacr.2022.02.041

12. DeGroff A, Gressard L, Glover-Kudon R, et al. Assessing the implementation of a patient navigation intervention for colonoscopy screening. *BMC Health Serv Res*. 2019;19(1):803. doi:10.1186/s12913-019-4601-4

13. Dudgeon D, King S, Howell D, et al. Cancer Care Ontario’s experience with implementation of routine physical and psychological symptom distress screening. *Psychooncology*. 2012;21(4):357-364. doi:10.1002/pon.1918

14. Duong HT, Hopfer S. “Let’s Chat”: process evaluation of an intergenerational group chat intervention to increase cancer prevention screening among Vietnamese American families. *Transl Behav Med*. 2021;11(3):891-900. doi:10.1093/tbm/ibaa120

15. Escoffery C, Riehman K, Watson L, et al. Facilitators and barriers to the implementation of the HPV VACs (Vaccinate Adolescents Against Cancers) Program: A consolidated framework for implementation research analysis. *Prev Chronic Dis*. 2019;16:E85. doi:10.5888/pcd16.180406

16. Friedman L, Engelking C, Wickham R, Harvey C, Read M, Whitlock KB. The EDUCATE Study: A continuing education exemplar for Clinical Practice Guideline Implementation. *Clin J Oncol Nurs*. 2009;13(2):219-230. doi:10.1188/09.CJON.219-230

17. Friese CR, Mendelsohn-Victor K, Ginex P, McMahon CM, Fauer AJ, McCullagh MC. Lessons learned from a practice-based, multisite intervention study with nurse participants. *J Nurs Scholarsh*. 2017;49(2):194-201. doi:10.1111/jnu.12279

18. Gesthalter YB, Koppelman E, Bolton R, et al. Evaluations of implementation at early-adopting lung cancer screening programs: Lessons learned. *Chest*. 2017;152(1):70-80. doi:10.1016/j.chest.2017.02.012

19. Gilleland Marchak J, Halpin SN, Escoffery C, Owolabi S, Mertens AC, Wasilewski-Masker K. Using formative evaluation to plan for electronic psychosocial screening in pediatric oncology. *Psychooncology*. 2021;30(2):202-211. doi:10.1002/pon.5550

20. Ginex PK, Arnal C, Ellis D, Guinigundo A, Liming K, Wade B. Translating evidence to practice: A multisite collaboration to implement guidelines and improve constipation management in patients with cancer. *Clin J Oncol Nurs*. 2021;25(6):721-724. doi:10.1188/21.CJON.721-724

21. Glaser KM, McDaniel DC, Rokitka DA, Hess SM, Flores TF, Reid ME. Implementing an integrative survivorship program at a comprehensive cancer center: A multimodal approach to life after cancer. *J Alt Complement Med*. 2019;25:S106-S111. doi:10.1089/acm.2018.0383

22. Gulasingam P, Haq R, Mascarenhas Johnson A, et al. Using implementation science to promote the use of the G8 screening tool in geriatric oncology. *J Am Geriatr Soc*. 2019;67(5):898-904. doi:10.1111/jgs.15920

23. Hämmerli P, Moukam AD, Wisniak A, et al. “My motivation was to save”: A qualitative study exploring factors influencing motivation of community healthcare workers in a cervical cancer screening program in Dschang, Cameroon. *Reprod Health*. 2022;19(1). doi:10.1186/s12978-022-01420-y

24. Haugen M, Kelly KP, Leonard M, et al. Nurse-led programs to facilitate enrollment to children’s oncology group cancer control trials. *J Pediatr Oncol Nurs*. 2016;33(5):387-391. doi:10.1177/1043454215617458

25. Hempstead B, Green C, Briant KJ, Thompson B, Molina Y. Community Empowerment Partners (CEPs): A breast health education program for African-American women. *J Community Health*. 2018;43(5):833-841. doi:10.1007/s10900-018-0490-4

26. Hohl SD, Bird JE, Nguyen CVT, et al. Operationalizing leadership and clinician buy-in to implement evidence-based tobacco treatment programs in routine oncology care: A mixed-method study of the U.S. Cancer Center Cessation Initiative. *Curr Oncol*. 2022;29(4):2406-2421. doi:10.3390/curroncol29040195

27. Hu Y, Byrne M, Archibald-Heeren B, et al. Implementing user-defined atlas-based auto-segmentation for a large multi-centre organisation: the Australian experience. *J Med Radiat Sci*. 2019;66(4):238-249. doi:10.1002/jmrs.359

28. Iwata AJ, Olden HA, Kippen KE, Swegal WC, Johnson CC, Chang SS. Flexible model for patient engagement: Achieving quality outcomes and building a research agenda for head and neck cancer. *Head Neck*. 2019;41(4):1087-1093. doi:10.1002/hed.25584

29. Kennedy ED, Milot L, Fruitman M, et al. Development and implementation of a synoptic MRI report for preoperative staging of rectal cancer on a population-based level. *Dis Colon Rectum*. 2014;57(6):700-708. doi:10.1097/DCR.0000000000000123

30. Knerr S, West KM, Angelo FA. Organizational readiness to implement population-based screening and genetic service delivery for hereditary cancer prevention and control. *J Genet Couns*. 2020;29(5):867-876. doi:10.1002/jgc4.1216

31. Lee H, Ho PS, Wang WC, Hu CY, Lee CH, Huang HL. Effectiveness of a health belief model intervention using a lay health advisor strategy on mouth self-examination and cancer screening in remote aboriginal communities: A randomized controlled trial. *Patient Educ Couns*. 2019;102(12):2263-2269. doi:10.1016/j.pec.2019.07.001

32. Lee SJC, Higashi RT, Inrig SJ, et al. County-level outcomes of a rural breast cancer screening outreach strategy: a decentralized hub-and-spoke model (BSPAN2). *Transl Behav Med*. 2017;7(2):349-357. doi:10.1007/s13142-016-0427-3

33. Leeman J, Askelson N, Ko LK, et al. Understanding the processes that Federally Qualified Health Centers use to select and implement colorectal cancer screening interventions: a qualitative study. *Transl Behav Med*. 2020;10(2):394-403. doi:10.1093/tbm/ibz023

34. Loo S, Mullikin K, Robbins C, et al. Patient navigator team perceptions on the implementation of a citywide breast cancer patient navigation protocol: a qualitative study. *BMC Health Serv Res*. 2022;22(1):683. doi:10.1186/s12913-022-08090-3

35. Lovell MR, Phillips JL, Luckett T, et al. Effect of cancer pain guideline implementation on pain outcomes among adult outpatients with cancer-related pain: A SteppedWedge Cluster randomized trial. *JAMA Netw Open*. 2022;5(2). doi:10.1001/jamanetworkopen.2022.0060

36. Mahloch J, Taylor V, Taplin S, Urban N. A breast cancer screening educational intervention targeting medical office staff. *Health Educ Res*. 1993;8(4):567-579. doi:10.1093/her/8.4.567

37. McBride K, Gesink D. Increasing cancer screening among old order Anabaptist women through specialized women’s health and integrated cancer screening interventions. *J Immigr Minor Health*. 2018;20(2):465-478. doi:10.1007/s10903-017-0551-2

38. Mittal N, Langevin AM, Kyono W, et al. Barriers to pediatric oncologist enrollment of adolescents and young adults on a cross-network national clinical trials network supportive care cancer clinical trial. *J Adolescent Young Adult Oncol*. 2022;11(1):117-121. doi:10.1089/jayao.2021.0041

39. Molina Y, Strayhorn SM, Bergeron NQ, et al. Navigated African American breast cancer patients as incidental change agents in their family/friend networks. *Supportive Care Cancer*. 2022;30(3):2487-2496. doi:10.1007/s00520-021-06674-z

40. O’Donovan B, Mooney T, Rimmer B, et al. Advancing understanding of influences on cervical screening (non)-participation among younger and older women: A qualitative study using the theoretical domains framework and the COM-B model. *Health Expect*. 2021;24(6):2023-2035. doi:10.1111/hex.13346

41. Payne D, Haith‐Cooper M, Almas N. “Wise up to cancer”: Adapting a community based health intervention to increase UK South Asian women’s uptake of cancer screening. *Health Soc Care Community*. 2022;30(5):1979-1987. doi:10.1111/hsc.13579

42. Phillips-Angeles E, Song L, Hannon PA, et al. Fostering partnerships and program success. *Cancer*. 2013;119(Suppl.15):2884-2893. doi:10.1002/cncr.28157

43. Rafie CL, Hauser L, Michos J, Pinsky J. Creating a workplace culture of preventive health: Process and outcomes of the Colon Cancer–Free Zone at Virginia Cooperative Extension. *J Cancer Educ*. 2020;35(6):1135-1140. doi:10.1007/s13187-019-01569-4

44. Rao SR, Salins N, Goh CR, Bhatnagar S. Building palliative care capacity in cancer treatment centres: a participatory action research. *BMC Palliat Care*. 2022;21(1):101. doi:10.1186/s12904-022-00989-2

45. Redwood D, Holman L, Zandman-Zeman S, Hunt T, Besh L, Katinszky W. Collaboration to increase colorectal cancer screening among low-income uninsured patients. *Prev Chronic Dis*. 2011;8(3):A69.

46. Rhode J, James S, Wheeler SB, et al. Facilitators and barriers of a health department-based mailed fecal testing program. *N C Med J*. 2020;81(5):293-299. doi:10.18043/ncm.81.5.293

47. Ribeiro RC, Antillon F, Pedrosa F, Pui CH. Global pediatric oncology: Lessons from partnerships between high-income countries and low- to mid-income countries. *J Clin Oncol*. 2016;34(1):53-61. doi:10.1200/JCO.2015.61.9148

48. Robinson TE, Janssen A, Harnett P, et al. Embedding continuous quality improvement processes in multidisciplinary teams in cancer care: Exploring the boundaries between quality and implementation science. *Aust Health Rev*. 2017;41(3):291-296. doi:10.1071/AH16052

49. Rocque GB, Partridge EE, Pisu M, et al. The patient care connect program: Transforming health care through lay navigation. *J Oncol Pract*. 2016;12(6):e633-e640. doi:10.1200/JOP.2015.008896

50. Rogers LQ, Goncalves L, Martin MY, et al. Beyond efficacy: A qualitative organizational perspective on key implementation science constructs important to physical activity intervention translation to rural community cancer care sites. *J Cancer Surviv*. 2019;13(4):537-546. doi:10.1007/s11764-019-00773-x

51. Schoenberg NE, Hatcher J, Dignan MB, Shelton B, Wright S, Dollarhide KF. Faith Moves Mountains: An Appalachian cervical cancer prevention program. *Am J Health Behav*. 2009;33(6):627-638. doi:10.5993/ajhb.33.6.1

52. Sendall MC, Stoneham M, Crane P, et al. Outdoor workers and sun protection strategies: Two case study examples in Queensland, Australia. *Rural Remote Health*. 2016;16(2):3558.

53. Sharma KP, DeGroff A, Maxwell AE, Cole AM, Escoffery NC, Hannon PA. Evidence-based interventions and colorectal cancer screening rates: The Colorectal Cancer Screening Program, 2015-2017. *Am J Prev Med*. 2021;61(3):402-409. doi:10.1016/j.amepre.2021.03.002

54. Sharma KP, Leadbetter S, DeGroff A. Characterizing clinics with differential changes in the screening rate in the Colorectal Cancer Control Program of the Centers for Disease Control and Prevention. *Cancer*. 2021;127(7):1049-1056. doi:10.1002/cncr.33325

55. Shelton RC, Dunston SK, Leoce N, Jandorf L, Thompson HS, Erwin DO. Advancing understanding of the characteristics and capacity of African American women who serve as lay health advisors in community-based settings. *Health Educ Behav*. 2017;44(1):153-164. doi:10.1177/1090198116646365

56. Simunovic M, Coates A, Smith A, Thabane L, Goldsmith CH, Levine MN. Uptake of an innovation in surgery: Observations from the cluster-randomized Quality Initiative in Rectal Cancer trial. *Can J Surg*. 2013;56(6):415-421. doi:10.1503/cjs.019112

57. Slatore CG, Golden SE, Thomas T, Bumatay S, Shannon J, Davis M. “It’s Really Like Any Other Study”: Rural radiology facilities performing low-dose computed tomography for lung cancer screening. *Ann Am Thorac Soc*. 2021;18(12):2058-2066. doi:10.1513/AnnalsATS.202103-333OC

58. Sopcak N, Aguilar C, O’Brien MA, et al. Implementation of the BETTER 2 program: A qualitative study exploring barriers and facilitators of a novel way to improve chronic disease prevention and screening in primary care. *Implement Sci*. 2016;11(1):158. doi:10.1186/s13012-016-0525-0

59. Stacey D, Taljaard M, Breau RH, et al. A patient decision aid for men with localized prostate cancer: A comparative case study of natural implementation approaches. *Cancer Nurs*. 2020;43(1):E10-E21. doi:10.1097/NCC.0000000000000651

60. Steinberg ML, Fremont A, Khan DC, et al. Lay patient navigator program implementation for equal access to cancer care and clinical trials: Essential steps and initial challenges. *Cancer*. 2006;107(11):2669-2677. doi:10.1002/cncr.22319

61. Szeszulski J, Craig DW, Walker TJ, Foster M, Mullen PD, Fernandez ME. Applying evidence-based intervention (EBI) mapping to identify the components and logic of colorectal cancer screening interventions. *Transl Behav Med*. 2022;12(2):304-323. doi:10.1093/tbm/ibab140

62. Thompson B, Coronado G, Chen L, Islas I. Celebremos La Salud! A community randomized trial of cancer prevention (United States). *Cancer Causes Control*. 2006;17(5):733-746. doi:10.1007/s10552-006-0006-x

63. Townsend JS, Pinkerton B, McKenna SA, et al. Targeting children through school-based education and policy strategies: Comprehensive cancer control activities in melanoma prevention. *J Am Acad Dermatol*. 2011;65(5,Suppl.1):S104-S13. doi:10.1016/j.jaad.2011.05.036

64. Truant TL, Green E, Ayala De Calvo LE, et al. Enhancing nurses’ oral therapy practice in 4 Latin American countries: A collaborative and participatory approach. *Cancer Nurs*. 2017;40(6):E49-E59. doi:10.1097/NCC.0000000000000434

65. Urquhart R, Kendell C, Geldenhuys L, et al. The role of scientific evidence in decisions to adopt complex innovations in cancer care settings: A multiple case study in Nova Scotia, Canada. *Implement Sci*. 2019;14(1):14. doi:10.1186/s13012-019-0859-5

66. Urquhart R, Porter GA, Sargeant J, Jackson L, Grunfeld E. Multi-level factors influence the implementation and use of complex innovations in cancer care: A multiple case study of synoptic reporting. *Implement Sci*. 2014;9:121. doi:10.1186/s13012-014-0121-0

67. Warner EL, Wu YP, Hacking CC, et al. An assessment to inform pediatric cancer provider development and delivery of survivor care plans. *J Cancer Educ*. 2015;30(4):677-684. doi:10.1007/s13187-015-0829-9

68. Watson L, Cotter MM, Shafer S, Neloms K, Smith RA, Sharpe K. Implementation of a lung cancer screening program in two federally qualified health centers. *Public Health Rep*. 2021;136(4):397-402. doi:10.1177/0033354920971717

69. Williams G, Mueller J, Mbeledogu C, et al. The impact of a volunteer-led community cancer awareness programme on knowledge of cancer risk factors and symptoms, screening, and barriers to seeking help. *Patient Educ Couns*. 2020;103(3):563-570. doi:10.1016/j.pec.2019.09.025

70. Williams KC, Brothers BM, Ryba MM, Andersen BL. Implementing evidence-based psychological treatments for cancer patients. *Psychooncology*. 2015;24(12):1618-1625. doi:10.1002/pon.3937

71. Williams LB, McCall A, Joshua TV, Looney SW, Tingen MS. Design of a community-based lung cancer education, prevention, and screening program. *West J Nurs Res*. 2019;41(8):1152-1169. doi:10.1177/0193945919827261

72. Williams LB, Shelton BJ, Gomez ML, Al-Mrayat YD, Studts JL. Using implementation science to disseminate a lung cancer screening education intervention through community health workers. *J Community Health*. 2021;46(1):165-173. Doi:10.1007/s10900-020-00864-2

73. Wright S, Porteous M, Stirling D, Young O, Gourley C, Hallowell N. Negotiating jurisdictional boundaries in response to new genetic possibilities in breast cancer care: The creation of an “oncogenetic taskscape.” *Soc Sci Med*. 2019;225:26-33. doi:10.1016/j.socscimed.2019.02.020

74. Yan A, Hooyer K, Asan O, Flower M, Whittle J. Engaging veteran stakeholders to identify patient-centred research priorities for optimizing implementation of lung cancer screening. *Health Expect*. 2022;25(1):408-418. doi:10.1111/hex.13401
